# Likelihood contrasts: a machine learning algorithm for binary classification of longitudinal data

**DOI:** 10.1038/s41598-020-57924-9

**Published:** 2020-01-23

**Authors:** Riku Klén, Markku Karhunen, Laura L. Elo

**Affiliations:** 1 Turku Bioscience Centre, University of Turku and Åbo Akademi University, Turku, Finland; 2 Turku PET Centre, University of Turku, Turku, Finland

**Keywords:** Computational models, Statistical methods

## Abstract

Machine learning methods have gained increased popularity in biomedical research during the recent years. However, very few of them support the analysis of longitudinal data, where several samples are collected from an individual over time. Additionally, most of the available longitudinal machine learning methods assume that the measurements are aligned in time, which is often not the case in real data. Here, we introduce a robust longitudinal machine learning method, named likelihood contrasts (LC), which supports study designs with unaligned time points. Our LC method is a binary classifier, which uses linear mixed models for modelling and log-likelihood for decision making. To demonstrate the benefits of our approach, we compared it with existing methods in four simulated and three real data sets. In each simulated data set, LC was the most accurate method, while the real data sets further supported the robust performance of the method. LC is also computationally efficient and easy to use.

## Introduction

Many biomedical studies consist of longitudinal data, i.e. data with multiple samples for each individual, taken at different time points. Here, we define longitudinal data so that the covariates are measured repeatedly, but not necessarily at even intervals or at the same time points for each individual. This type of data turns out to yield substantial modelling challenges. For example, the most widely used binary classifiers, such as Lasso^1^, random forest^2^ and artificial neural networks^3^, are not designed for this type of data. Therefore, they cannot fully benefit from the repeated measurements. Moreover, those machine learning methods which support longitudinal data typically assume that the time points are aligned between the individuals^4^.

Many statistical methods are available for longitudinal data, especially within the discipline of econometrics^5^, but these methods typically also assume the time points to be aligned and evenly spaced. The only main exception suitable for biomedical data is the linear mixed-effects model (LME) and its modifications^6–9^, which support data with non-aligned time points. However, the LME model is a regression model for a continuous response variable. Many different solutions to turn the model into binary classifier can be envisaged^10,11^. Here, we present one such solution: the method of likelihood contrasts (LC). We introduce this novel method because it exploits all longitudinal data in classification instead of a single time point or average. LC is fast and easy to calculate, and secondly, our results show its good performance in simulated and real data sets alike.

We take the univariate LME as the starting point and use it as a building block for our LC algorithm. Briefly, we fit LMEs using a standard software package (lme4 version 3.1–131.1)^12^, and then use their maximised log-likelihood functions for inference. We assign each sample to the group where the log-likelihood changes most favourably. Thus, this method amounts to a binary classifier. However, contrary to many other machine learning methods, LC is computationally very efficient, easy to implement and the need to fine-tune parameters is minimal. We provide an open-source implementation of LC at https://elolab.utu.fi/software/.

In this paper, we demonstrate the performance of LC in four simulated and three publicly available real data sets. In each data set, we test the discriminatory power of LC regarding the case-control status of the study subjects and compare it to that of widely used machine learning and predictive algorithms, including Lasso^1^, random forest (RF)^2^, support vector machines (SVM)^13^, neural networks (NN)^3^, and LME regression models. Two of the real data sets derive from the book of Rizopoulos^14^ on longitudinal models and are openly accessible^15^. These represent typical clinical data sets used for longitudinal modelling. The third real data set concerns molecular data on pediatric Type 1 Diabetes mellitus collected in the Finnish Type 1 Diabetes Prediction and Prevention (DIPP) study^16^ and is also publicly available^17^.

## Methods

In this section, we introduce the method of likelihood contrasts (LC). We also describe other methods used in comparison.

### Likelihood contrasts

Let ${z}_{i}$ denote an observation of a new individual *i*, which may contain data from multiple time points, and response variables and covariates alike. The individuals are labelled as ${\delta }_{i}=1$ (case) or ${\delta }_{i}=0$ (control). We estimate two separate models and see which one gives ${z}_{i}$ a better fit. To this end, let ${z}_{-i}^{1}$ and ${z}_{-i}^{0}$ denote training data from cases and controls, respectively, and let $\ell ({z}_{-i}^{1}|{\theta }^{1})$ and $\ell ({z}_{-i}^{0}|{\theta }^{0})$ denote the maximised log-likelihoods of the corresponding two separate models, *M*^1^ and *M*^0^, with the parameter estimates *θ*^1^ and *θ*^0^. We then calculate the likelihood contrasts as

1 $$\begin{array}{ccc}{d}_{1} & = & \ell ({z}_{i},{z}_{-i}^{1}|{\theta }^{1})-\ell ({z}_{-i}^{1}|{\theta }^{1}),\\ {d}_{0} & = & \ell ({z}_{i},{z}_{-i}^{0}|{\theta }^{0})-\ell ({z}_{-i}^{0}|{\theta }^{0})\end{array}$$

and assign ${z}_{i}$ to the group where ${d}_{k}$ is larger.

It is also possible to extend the method to yield probability scores. This can be justified by considering the log-likelihood difference ${d}_{1}-{d}_{0}$. Quite intuitively, ${d}_{1}-{d}_{0}=0$ can be used as a cut-off point in binary classification. In this respect, ${d}_{1}-{d}_{0}$ is comparable to the linear predictor (i.e., the linear combination of covariates) found in logit models. In logit models, the linear predictor is mapped to probability score through the inverse of the logistic link function. Adopting this approach, we have

2 $$\hat{P}({\delta }_{i}=1)={{\rm{logit}}}^{-1}({d}_{1}-{d}_{0})=\frac{\exp ({d}_{1}-{d}_{0})}{1+\exp ({d}_{1}-{d}_{0})}=\frac{\exp ({d}_{1})}{\exp ({d}_{0})+\exp ({d}_{1})}.$$

It is quite naturally possible to extend LC into a multinomial classifier. In that case,

3 $$\begin{array}{rcl}{d}_{k} & = & \ell ({z}_{i},{z}_{-i}^{k}|{\theta }^{k})-\ell ({z}_{-i}^{k}|{\theta }^{k}),\,k=1,\ldots ,L,\\ {\hat{\delta }}_{i} & = & \text{arg}\mathop{\max }\limits_{k}{d}_{k}\end{array}$$

and

4 $$\hat{P}({\delta }_{i}=k)=\frac{\exp ({d}_{k})}{{\sum }_{l=1}^{L}\exp ({d}_{l})},k=1,\ldots ,L,$$

where *L* is the number of classes. However, we only use binary classification in this paper.

### Implementation with mixed models

Above, ${z}_{i}$ denotes any data, encompassing ${n}_{i}$ measurements for individual *i*. Here, we use LC in combination with LMEs, which are versatile tools for modelling jointly the effect of covariates, confounders and sampling artefacts. In matrix notation, an LME can be defined as

5 $$\begin{array}{c}{\boldsymbol{y}}={\bf{X}}{\boldsymbol{\beta }}+\epsilon \\ E(\epsilon |{\bf{X}})=0,\\ Cov(\epsilon |{\bf{X}})={\bf{A}}({\bf{X}}),\end{array}$$

where **y** is the vector of responses, **X** is the matrix of covariates, ***β*** is the vector of regression coefficients, and $\epsilon$ is the error term. ${\bf{A}}({\bf{X}})$ is the covariance matrix of the measurement errors which may depend on **X**. An LME differs from the usual linear model because ${\bf{A}}({\bf{X}})$ is not a diagonal matrix. The off-diagonal terms of ${\bf{A}}({\bf{X}})$ represent correlations between the samples, and are considered as the ‘mixed effects’. They arise as a result of study design or spatio-temporal vicinity of the samples.

In practice, we assume that the data for each individual *i* involve a disease-specific longitudinal marker ${y}_{ij}$ and a number of covariates denoted by ${{\boldsymbol{x}}}_{ij}$. In the sequel, we denote the *j* th measurement of a marker for individual *i* as *y*_*ij*_, and we model the time course of this marker as

6 $$\begin{array}{ccc}{y}_{ij} & = & {{\boldsymbol{\beta }}}_{1}^{\text{'}}{{\boldsymbol{x}}}_{ij}{t}_{ij}+{\gamma }_{1}{t}_{ij}+{{\boldsymbol{\theta }}}_{1}^{\text{'}}{{\boldsymbol{x}}}_{ij}+{u}_{i}+{{\epsilon }}_{ij},\,{\delta }_{i}=1,\\ {y}_{ij} & = & {{\boldsymbol{\beta }}}_{0}^{\text{'}}{{\boldsymbol{x}}}_{ij}{t}_{ij}+{\gamma }_{0}{t}_{ij}+{{\boldsymbol{\theta }}}_{0}^{\text{'}}{{\boldsymbol{x}}}_{ij}+{u}_{i}+{{\epsilon }}_{ij},\,{\delta }_{i}=0,\end{array}$$

where *δi* denotes the case-control status, ${t}_{ij}$ denotes time, ${u}_{i} \sim NID(0,{\sigma }_{i}^{2})$ denotes an individual-specific random effect and ${{\epsilon }}_{ij} \sim NID(0,{\sigma }^{2})$ denotes a measurement error. Here, $NID(\mu ,{\sigma }^{2})$ means normal, independent and identically distributed with mean *μ* and variance σ^2^. There are two models, as there are two groups of patients (${\delta }_{i}=1$ and ${\delta }_{i}=0$). The purpose of LC is to distinguish between these groups. An LME-based LC algorithm is implemented in R and is publicly available at https://elolab.utu.fi/software/.

### Connection to statistical paradigms

The decision rule of LC resembles the likelihood-ratio (LR) test and Bayesian posterior probabilities but differs from both. In this subsection, we discuss these similarities and differences. Generally speaking, as compared to LR test, our method is not based on nested models, and is thus more general. As compared to Bayesian inference, our method does not require numerical integration schemes, and is thus more efficient. Moreover, our method is suited for situations where the likelihood contribution of each individual observation cannot be calculated, as it uses the change of log-likelihood as a proxy for likelihood contribution.

In more detail, the LR test statistic is defined as

7 $$LR=2(\ell ({y}_{i},{y}_{-i}^{1},{y}_{-i}^{0}|{\theta }^{1})-\ell ({y}_{i},{y}_{-i}^{1}{y}_{-i}^{0}|{\theta }^{0})),$$

This definition is very standard and can be found from statistics text books. It is based on the fact that the likelihood ratio thus defined has favourable distributional properties known as the Wilks’ theorem. The LR test, however, is based on nested models. For our method, the implied test statistic is

8 $${d}_{1}-{d}_{0}=\ell ({y}_{i},{y}_{-i}^{1}|{\theta }^{1})-\ell ({y}_{i},{y}_{-i}^{0}|{\theta }^{0})-\ell ({y}_{-i}^{1}|{\theta }^{1})+\ell ({y}_{-i}^{0}|{\theta }^{0})$$

and it can be calculated for disjoint models. In parallel with this, Bayesian posterior probabilities typically concern the whole data, as the purpose is to choose the optimal model. Contrary to this, LC concerns each observation separately. To see the connection to Bayesian inference, assume that the information in ${y}_{-i}^{0}$ and ${y}_{-i}^{1}$ is so great that it essentially fixes the values of *θ*^0^ and *θ*^1^. Following this,

9 $$\ell ({y}_{i}|{\theta }^{k})=\,\log \,\pi ({y}_{i}|{M}^{k}),\,k=0,1;$$

i.e. the maximised likelihood is the likelihood of the whole model. (In other cases, one would need to integrate over *θ*^*k*^ to get $\pi ({y}_{i}|{M}^{k})$; see Gelman *et al*.^18^.) Moreover, let us assume that the observation units are independent, and thus, the likelihood is separable as

10 $$\ell ({y}_{i},{y}_{-i}^{k}|{\theta }^{k})=\ell ({y}_{i}|{\theta }^{k})+\ell ({y}_{-i}^{k}|{\theta }^{k}).$$

Thus, it follows that

11 $$\log \,\pi ({y}_{i}|{M}^{k})=\ell ({y}_{i}|{\theta }^{k})=\ell ({y}_{i},{y}_{-i}^{k}|{\theta }^{k})-\ell ({y}_{-i}^{k}|{\theta }^{k})={d}_{k}.$$

Now, if one gives equal prior weights for both models, i.e. $\pi ({M}^{0}),\pi ({M}^{1})=1/2$, it follows that

12 $$\pi ({M}^{1}|{y}_{i})=\frac{\pi ({y}_{i}|{M}^{1})\pi ({M}^{1})}{\pi ({y}_{i}|{M}^{0})\pi ({M}^{0})+\pi ({y}_{i}|{M}^{1})\pi ({M}^{1})}=\frac{\exp ({d}_{1})}{\exp ({d}_{0})+\exp ({d}_{1})},$$

i.e. the posterior probability of *M*^1^ for ${y}_{i}$ coincides with our probability score, see Eq. (2). Finally, someone might ask why we use the likelihood contrasts *d*_0_ and *d*_1_ to classify individual *i*, and not just the likelihood contributions $\ell ({y}_{i}|{\theta }^{0})$ and $\ell ({y}_{i}|{\theta }^{1})$. This is because in complex models, such as LME, it is not possible to calculate likelihood contributions as such. Thus, we use the likelihood contrast

13 $${d}_{k}=\ell ({y}_{i},{y}_{-i}^{1}|{\theta }^{k})-\ell ({y}_{-i}^{1}|{\theta }^{k})$$

as a proxy for $\ell ({y}_{i}|{\theta }^{k})$. Note that LC does not produce a single model with fixed coefficients, but it creates new coefficients for each new individual.

### Comparison with other methods

We compared the performance of LC to a number of statistical and machine learning methods, including LME, linear feature extraction (LF), logit mixed-effects regression (implemented as the function GLMER in the R package lme4^19^), Lasso, random forests (RF), support vector machines (SVM), and neural networks (NN). Among the compared methods, LME and GLMER represent statistical methods, while Lasso, RF, SVM and NN are widely used machine learning algorithms. LF uses a strategy to account for the longitudinal dimension, but relies on a standard statistical technique, logistic regression. Below, we briefly outline these methods using the notations given above. In all analyses, the task was to predict ${\delta }_{i}$ on the basis of the covariates ${{\boldsymbol{x}}}_{ij}$ and the longitudinal marker ${y}_{ij}$. We denote the averaged value of the longitudinal marker over time by ${\bar{y}}_{i}$ and averaged covariates by ${{\boldsymbol{x}}}_{i}$.

In LME, the marker was first modelled as

14 $${\hat{y}}_{ij}={{\boldsymbol{\beta }}}^{\text{'}}{{\boldsymbol{x}}}_{ij}{t}_{ij}+\gamma {t}_{ij}+{{\boldsymbol{\theta }}}^{\text{'}}{{\boldsymbol{x}}}_{ij}+{u}_{i}+{{\epsilon }}_{ij},$$

where ${u}_{i} \sim NID(0,{\sigma }_{1}^{2})$ denotes an individual-specific random effect, and ${{\epsilon }}_{ij} \sim NID(0,{\sigma }_{2}^{2})$ denotes a measurement error. Then, we averaged the estimated values ${\hat{y}}_{ij}$ over time $j=1,\ldots ,{n}_{i}$ and ran a logit regression of ${\delta }_{i}$ on the averages. We fitted the LME models using the R package nlme version 3.1–131.1^12^.

In LF, we first ran a linear regression of ${y}_{ij}$ on ${t}_{ij}$ within each individual $i$ to obtain individual-specific slopes and intercepts, denoted by ${b}_{i}$ and ${a}_{i}$, respectively,

15 $${\hat{y}}_{ij}={b}_{i}{t}_{ij}+{a}_{i}.$$

Subsequently, we ran a logit regression of ${\delta }_{i}$ on $({{\boldsymbol{x}}}_{i},{b}_{i},{a}_{i})$ to predict ${\delta }_{i}$.

Logistic Lasso was fitted on $({\delta }_{i};{{\boldsymbol{x}}}_{i},{\bar{y}}_{i},{\bar{t}}_{i})$ using the R package glmnet version 2.0–13^20^ with ten-fold cross-validation. Here ${\bar{t}}_{i}$ is the averaged measurement time for each individual.

The GLMER model was constructed on $({\delta }_{i};{{\boldsymbol{x}}}_{ij},{y}_{ij},{t}_{ij})$ using the R package lme4 version 1.1–15^19^.

The RF model was constructed on $({\delta }_{i};{{\boldsymbol{x}}}_{i},{\bar{y}}_{i},{\bar{t}}_{i})$ using the R package randomForest version 4.6–12^21^.

An SVM (more precisely, epsilon regression) was constructed on $({\delta }_{i};{{\boldsymbol{x}}}_{i},{\bar{y}}_{i},{\bar{t}}_{i})$ using the R package e1071 version 1.6–8^22^.

An artificial neural network was constructed with one hidden layer on $({\delta }_{i};{{\boldsymbol{x}}}_{i},{\bar{y}}_{i},{\bar{t}}_{i})$ using the R package nnet version 7.3–12^23^.

Methods LME, Lasso, RF, SVM and NN involved averaging of values before modelling. We also implemented the methods without averaging by considering each time point as a separate measurement. We denote these methods by LME2, Lasso2, RF2, SVM2 and NN2.

All machine learning models were built using default parameters. Internal cross validation was used to determine coeffiecients for the logistic model and the penalty factor in Lasso. RF implemented Breiman’s random forest algorithm using 500 trees with sample replacement. In SVM, support vectors were defined using epsilon regression with $\varepsilon =0.1$. NN used one hidden layer and the number of units in the hidden layer was determined to be half of the number of variables.

Note that methods LC, LF, LME, LME2, Lasso2, RF2, SVM2, NN2 and GLMER use information for each time point, while methods Lasso, RF, SVM and NN use information averaged over time points per subject. Out of the compared methods, only LC, LF and GLMER directly make prediction for a new subject, while the other methods create a prediction for a single time point. For these methods, we made predictions for each time point for each subject, and averaged the predictions to obtain a single prediction for each subject.

### Model evaluations

We compared the performance of the different methods on the basis of their binary predictions for test data, using cross validations as explained in the sequel. We truncated the probability scores given by the different models into binary predictions by using 0.50 probability as the cut-off and then assessed the performance of the binary predictions by calculating sensitivity and specificity. We considered 0.50 as the baseline value of sensitivity and specificity, assuming that a completely uninformative classifier is equally likely to classify the subjects as cases or controls. We used Wilcoxon’s rank sum test to compare the sensitivity and specificity obtained from each method to the baseline values. Different methods were compared using paired Wilcoxon’s rank sum test. To account for multiple testing, we applied Benjamini-Hochberg false discovery rate (FDR) correction^24^ to the Wilcoxon’s rank sum test P-values.

Additionally, we also present the F1 scores, accuracies and receiver operating characteristic (ROC) curves for each method and data set in Supplementary material.

## Materials

To evaluate LC along with existing predictive methods we used simulated and real data.

### Simulated data

In the simulated data, we considered one static covariate, the ‘treatment’ denoted by ${x}_{i}$, and one longitudinal marker denoted by ${y}_{ij}$. We assumed here that the distributional form of the marker differed between cases and controls, and thus, ${y}_{ij}$ was informative regarding the case-control label ${\delta }_{i}$. Altogether, we considered four different scenarios described in detail below.

In each scenario, the individuals were equally likely to be cases or controls. We assumed four time points per individual (${n}_{i}=4$) and we assumed ${x}_{i}$ to be Bernoulli distributed with parameter value 0.5 and ${t}_{ij}$ to be uniformly distributed on the interval $(-1,1)$, i.e. we assumed that the treatment was allocated randomly and the time was measured relative to the event. In each scenario, 1,000 replicate data sets were generated to control for the sampling variation.

In Scenario 1, we assumed that the cases and controls reacted differently to the treatment and also that the natural course of the marker was different between the groups. Thus, we specified the model as

16 $$\begin{array}{ccc}{y}_{ij} & = & {x}_{i}-{t}_{ij}+{u}_{i}+{\varepsilon }_{ij},\,{\delta }_{i}=0,\\ {y}_{ij} & = & -{x}_{i}+{t}_{ij}+{u}_{i}+{\varepsilon }_{ij},\,{\delta }_{i}=1,\end{array}$$

where ${u}_{i} \sim NID(0,0.25)$ is an individual-specific random effect and ${\varepsilon }_{ij} \sim NID(0,0.25)$ is the measurement error. Here, as in Eq. (17) below, the coefficients were chosen to illustrate the biological phenomena explained in the text, simultaneously keeping the simulation model as simple and tangible as possible. In this scenario, we used ${n}_{1}=40$ samples as the training data and assessed the model performance in an independent test data of ${n}_{2}=20$ samples, repeating the process 1,000 times.

In Scenario 2, we assumed that the distribution of ${y}_{ij}$ was more similar between the cases and controls than in Scenario 1. We assumed that the controls did not react to the treatment and the natural course of the marker was similar between the groups, albeit milder in controls. Thus, we specified the model as

17 $$\begin{array}{ccc}{y}_{ij} & = & 0.5{t}_{ij}+{u}_{i}+{\varepsilon }_{ij},\,{\delta }_{i}=0,\\ {y}_{ij} & = & -{x}_{i}+{t}_{ij}+{u}_{i}+{\varepsilon }_{ij},\,{\delta }_{i}=1,\end{array}$$

where ${u}_{i} \sim NID(0,0.5)$ is an individual-specific random effect and ${\varepsilon }_{ij} \sim NID(0,0.5)$ is the measurement error. Also in this scenario, we used ${n}_{1}=40$ samples as the training data and assessed the model performance in an independent test data of ${n}_{2}=20$ samples, repeating the process 1,000 times.

Scenarios 3 and 4 were the same as Scenarios 1 and 2, respectively, but here we assumed larger training and test data sets with ${n}_{1}=160$ and ${n}_{2}=80$.

### Real data

We used two clinical data sets and one high-throughput molecular data set. In each of these real data sets, we used 2/3 of the data set as training data and predicted the labels in the remaining 1/3 to assess the performance of the different methods. To control for sampling variation in model evaluation, we repeated the process 1,000 times which diminished the standard errors more than sufficiently (<0.01 for variables on a scale of 0–1).

### Clinical data sets

The two clinical data sets (Pbc2 and Prothro) were chosen from Rizopoulos^14^, distributed in the R package JM (version 1.4–7)^15^. The sample sizes of the real data sets are summarised in Table 1. In both clinical data sets, ${\delta }_{i}=1$ means death and ${\delta }_{i}=0$ staying alive.

**Table 1:** Sample sizes of the real data sets.

	Total number of samples (*N*)	Number of individuals (*n*)	Number of cases	Number of controls	Number of time points per individual ± SD
Pbc2	1,945	312	140	172	6.2 ± 3.8
Prothro	2,968	488	292	196	6.1 ± 3.5
DIPP, seroconverted	68	17	4	13	4.0 ± 0.0
DIPP, progressor	238	40	18	22	6.0 ± 1.9

The Pbc2 data set was from a study on primary biliary cirrhosis^25^. The longitudinal marker in this data set was logarithm of blood bilirubin (mg/dl) over time and we used the drug (placebo or D-penicillamine) as a static covariate. Time (years) and bilirubin values were scalar numbers and drug status had a binary value.

The Prothro data set was from a study of liver cirrhosis^26^. The longitudinal marker was prothropin level and we used the treatment (placebo or prednisone) as a static covariate. Time (years) and prothrombin levels were scalar numbers, and treatment status was a binary variable.

For Pbc2 and Prothro, we used the LME regression (in methods LC and LME) motivated by Rizopoulos^14^ as

18 $${y}_{ij}=\alpha +\beta {x}_{i}{t}_{ij}+\gamma {t}_{ij}+\theta {x}_{i}+{u}_{i}+{{\epsilon }}_{ij},$$

where ${x}_{i}$ denotes the medication status of individual $i$.

### High-throughput molecular data set

The high-throughtput molecular data set was from the Finnish Type 1 Diabetes Prediction and Prevention (DIPP) study^16^ and involved preprocessed mRNA expression levels measured on Affymetrix Human Genome U219 microarray^17^. There was a total of 49,386 probes corresponding to different genes in the data that were measured over time. The data were downloaded from The National Center for Biotechnology Information webpage (https://www.ncbi.nlm.nih.gov/) using Gene Expression Omnibus identifier GSE30211. Each probe was z-scored. The mRNA data consisted of two separate data sets: seroconverted children and progressors. The data set of seroconverted children contained samples from subjects close to the time when diabetes-related autoantibodies developed. Samples of the progressors’ data set were concentrated close to the diagnosis of diabetes. Here, we focused on the clinical phenotype, i.e. progressors, and defined the case-control label as diagnosed (${\delta }_{i}=1$) or not diagnosed (${\delta }_{i}=0$) with Type 1 diabetes.

To select the probes to be used as the longitudinal covariates, we first used the data from seroconverted children. For each seroconverted child, the first four follow-up samples were selected. Probes with median expression lower than the median of median expressions (5.47) were excluded. The remaining 24,693 probes were ranked in two ways: 1., a ranking based on Wilcoxon’s rank sum test P-value between cases and controls for all samples, and 2., a ranking based on Wilcoxon’s rank sum test P-value between cases and controls for subjectwise median values. The two rankings were combined by taking the average rank and top five probes were selected. The selected probes were 11751509_a_at, 11723996_a_at, 11759536_a_at, 11733701_a_at and 11748922_x_at, and they mapped to genes *RCN1*, *GLCCI1*, *TTC17*, *FKBP11* and *NSMF*, respectively. For simplicity, we will refer to the probes by using their gene symbols. No static covariates were used.

To construct the predictive models we used the data set progressors. For methods LC and LME, we used age as marker ${y}_{ij}$ and top 5 probes as longitudinal covariates ${{\boldsymbol{x}}}_{ij}$ as

19 $${y}_{ij}=\alpha +{{\boldsymbol{\theta }}}^{\text{'}}{{\boldsymbol{x}}}_{ij}+{u}_{i}+{{\epsilon }}_{ij}.$$

For the other methods, we trained the models by using averaged information from the 5 top probes and age.

## Results

In this section, we represent the results for four scenarios of simulated data and three real data sets. In the simulated data, we considered one static covariate and one longitudinal marker. The four simulation scenarios differed in the distributions of the markers and in sample sizes. The real data sets contained two clinical data sets and one high-throughput molecular data set. We emphasise that we used multiple simulation replicates for simulated data, and exhaustive cross validation for real data.

We compared the performance of LC to a number of statistical and machine learning methods, including LF, LME, GLMER, Lasso, RF, SVM and NN, in terms of their sensitivity and specificity. Results for the methods LME2, Lasso2, RF2, SVM2 and NN2 are collected in the Supplementary material. Additionally, we present the F1 scores, accuracies and the receiver operating characteristic (ROC) curves for each method in the Supplementary material.

### Simulated data

**Figure 1:**
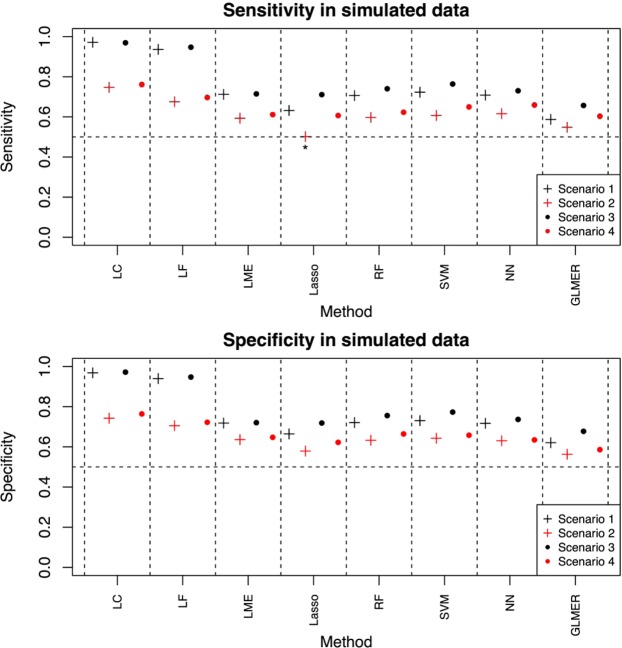
Model performance in simulated data. This figure represents sensitivity and specificity in simulated data. The Scenarios refer to data-generating process. The vertical lines around the dots represent standard error, when visible. Sensitivity and specificity have been calculated from 1,000 Monte Carlo replicates. Statistics *not* significant at the false discovery rate level of 0.05 have been indicated by asterisk (*).

In the simulated data, all methods had fairly good performance (Fig. 1, Supplementary Figs. 1–3, and Supplementary Table 1). All other combinations of methods and scenarios had highly significant sensitivity (P < 1.0 × 10^−6^) and specificity (P < 1.0 × 10^−6^) compared to the baseline value 0.5, except for sensitivity of Lasso in Scenario 2 (P = 0.90). Thus, it seems that Lasso was not able to distinguish between the cases and controls on the basis of the overlapping distributions of the temporal averages. In all scenarios, LC was the best method in terms of both sensitivity (in each pairwise comparison P < 1.0 × 10^−6^) and specificity (in each pairwise comparison P < 1.0 × 10^−6^), closely followed by LF. The F1 scores, accuracies and ROC curves supported similar conclusions (see Supplementary Table 1, and Supplementary Figs. 2 and 3).

The simulated scenarios differed from each other so that Scenarios 1 and 3 had greater distinction between cases and controls than Scenarios 2 and 4. On the other hand, Scenarios 3 and 4 had more samples than Scenarios 1 and 2. As expected, most methods achieved the best results in Scenarios 3 and 4, which had higher numbers of training samples (Fig. 1, Supplementary Fig. 1). However, for some methods (LC and LME), the difference between Scenarios 1 and 3, i.e. a difference attributable to sample size, was very small. Regarding the effect of the sample distributions, better results were achieved in Scenario 1 than in Scenario 2, as Scenario 2 had a smaller difference between the sample groups. A similar observation holds for Scenarios 3 and 4, as expected (Fig. 1, Supplementary Fig. 1). Methods LME2, Lasso2 and RF2 had similar performance compared to the corresponding averaged methods LME, Lasso and RF. In Scenarios 1 and 3, methods SVM2 and NN2 outperformed SVM and NN.

To conclude, the results obtained from the simulated data sets demonstrated that all methods could deliver meaningful results, and LC had a very good performance, as compared to the twelve other contemporary approaches tested.

### Real data

**Figure 2:**
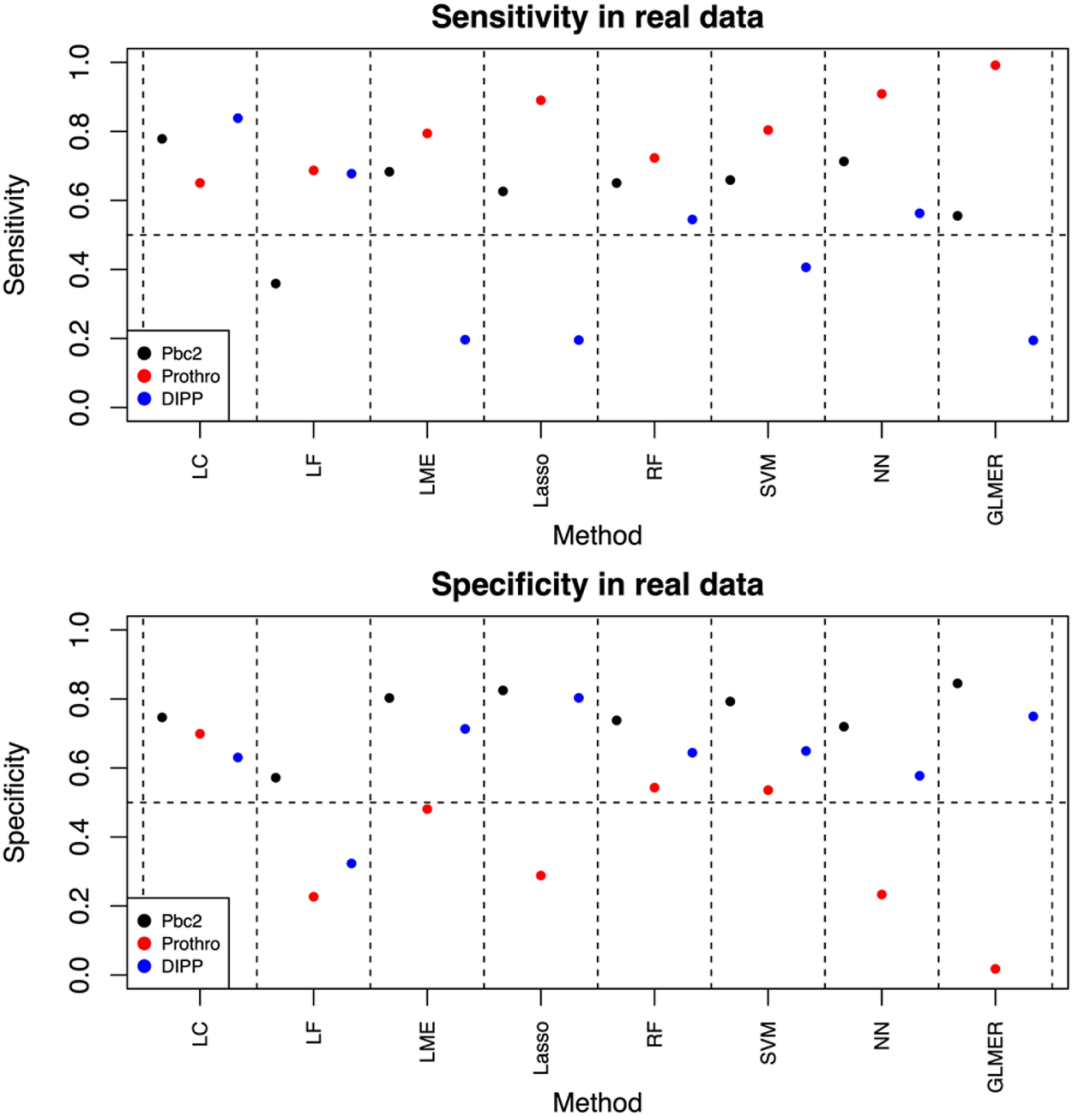
Model performance in real data. Pbc2 and Prothro are clinical data sets, whereas DIPP is a molecular data set from Type 1 Diabetes. The vertical lines around the dots represent standard error, when visible. Sensitivity and specificity have been calculated by using exhaustive cross validation, thus the small standard errors.

Although all the methods tested here performed well in the simulated data sets, this pattern changed when we moved to the real data sets (Fig. 2, Supplementary Figs. 4–6, and Supplementary Table 2). While LC and RF had both specificity and sensitivity highly significantly over 0.50 in all three real data sets (P < 10^−6^), this was not the case for any of the other methods. Instead, all the other methods had either sensitivity or specificity below 0.55 in at least one data set. The Prothro and DIPP data sets turned out to be the hardest to predict in terms of sensitivity and specificity. In the Prothro data, LC achieved sensitivity of 0.65 and specificity of 0.70, and in the DIPP data, sensitivity of 0.84 and specificity of 0.63. The relative difficulty of the real data sets was also seen in the F1 values, accuracies and ROC curves (Supplementary Table 2, Supplementary Figs. 5 and 6). LC was the only method that obtained accuracy and F1 value higher than 0.7 in all real data sets (Supplementary Table 2). In all real data sets, methods LME2, Lasso2, RF2, SVM2 and NN2 were slightly outperformed by the corresponding averaged methods.

Based on these results, one may conclude that LC was the only robust method among the tested thirteen, as it did not fail in any data set analysed in this study.

## Discussion and Conclusions

In this study, we examined thirteen methods for binary classification of longitudinal data with non-aligned time points, which is a common scenario in biomedical studies. (Most of these methods needed to be adjusted on an ad-hoc basis to acknowledge for the longitudinal nature of the data, i.e. we used temporal averages of covariates. However, this was not the case for our method.) We introduced the method of likelihood contrasts (LC) and compared its performance to the twelve other approaches, using simulated data and three real data sets. In the simulated data sets, LC clearly outperformed the other methods in terms of sensitivity and specificity (P < 1.0 × 10^−6^). In the real data sets, the performance of all methods was lower than in the simulated data. However, unlike most of the other methods, LC provided reasonable classification performance also in all the real data sets (specificity and sensitivity significantly over 0.50, P < 10^−6^), demonstrating its robustness over the other methods.

Another benefit of LC is that it can be generalised to any analysis scenario consisting of two models and a measure of model fit. For example, we used the difference of log-likelihood to measure the agreement between a new observation and the pre-existing data. In a non-parametric setting, one could use some other measure of model fit, such as the difference of mean squared errors.

Presently, we have used LC in combination with two LMEs. This derives from previous modelling tradition for longitudinal data^6,7^, but could also be changed. For example, the log-likelihood could be derived from a non-linear regression with time. However, a benefit of the LME framework is that it is fairly general, and the theory of these models is well-known. Moreover, the LME framework can easily be extended to allow for a wider range of applications. For example, it is possible to use penalised random-effects models for automated model choice^4^.

There are multiple studies comparing different binary classifiers for biomedical single time-point data. For example, Khondoker *et al*.^27^ compared four classification methods using simulated and real data sets. They concluded that linear discriminant analysis gave the best results for small data sets and SVM for data sets with more than 20 samples. Johnson *et al*.^28^ compared six classifiers in RNA-seq data. They found random forest to be the best method, and transcript-level data to be better suited for classification than gene-level data. Babu *et al*.^29^ studied the effect of feature selection for classification methods in cancer. They found that feature selection substantially improved the performance of classification, and in their study, SVM was one of the best methods together with Relief-F^30^ and information gain^31^.

Given the prevalence of longitudinal data sets in biomedicine, it is surprising that there are so few longitudinal binary classifiers. Longitudinal time-series experiments using DNA microarrays have already been performed for more than a decade^32–34^. In line with this, various statistical methods have been developed to detect the differentially expressed genes between experimental groups in the longitudinal data. For example, MaSigPro^32^ can be used to analyse inter-group differences by fitting polynomials of various degrees to expression data. The moderated F-test in limma^35^ can be used to discover differentially expressed genes by considering intergroup contrasts at different time points. Approaches relying on Bayesian statistics for detecting longitudinal differential gene expression have also been developed^33,34^. However, none of these methods directly addresses the question of classifying the longitudinal samples in two distinct groups, e.g. in patients and healthy controls. This is a shortcoming which we try to address by the proposed LC method.

Regarding binary classification of longitudinal data, there are some methods which operate on aligned time points^4^. However, a fully flexible model such as LC has not been developed before. Consequently, it is difficult to judge the performance of LC against a pre-existing baseline. Furthermore, regarding binary classification in static settings, earlier studies have not been able to highlight a single generally best method. For example, Pirooznia *et al*.^36^ used eight machine learning algorithms in eight microarray gene expression data sets, and they found SVM to have the best performance. In line with this, Castillo *et al*.^37^ analysed RNA-sequencing and microarray data sets, finding SVM to be more accurate than RF or nearest-neighbour classification. However, Bienkowska *et al*.^38^ used SVM and RF in combination with iterated feature selection in gene expression data. They found RF to outperform SVM in three out of four cases, in terms of area under the ROC curve. Such comparisons are numerous and can be found in many application areas^39,40^.

The two real clinical data sets used in our study were taken from Rizopoulos^14^. As our primary purpose was to compare the performance of the different classifiers, we used the same covariates and markers as the earlier studies^14^. In the Type 1 Diabetes data^17^, the choice of the predictive markers was less obvious. In these high-throughput molecular data, there were initially 49,386 measured probes. Following observations from previous studies, we filtered these features. For example, Pirooznia *et al*.^36^ have reported that up to 10% losses in predictive accuracy can be expected, if all features are used in place of an optimal feature set.

To conclude, results obtained in this study suggest that LC can be used as an accurate binary classifier in longitudinal data. LC outperformed the conventional machine learning methods in the simulated data. Although the three real data sets proved to be more difficult to predict correctly than the simulated data, LC was able to deliver statistically significant predictions in all data sets.

## Supplementary information

Supplementary information.

## Data Availability

The data sets generated during and/or analysed during the current study are available from the corresponding author on reasonable request.
