# Supplementary material for: Likelihood contrasts: a machine learning algorithm for binary classification of longitudinal data
Source: Sci Rep. 2020 Jan 23;10:1016. doi: 10.1038/s41598-020-57924-9 (PMC6978422; doi:10.1038/s41598-020-57924-9)
Supplement: Supplementary file 1 — Supplementary information. [file 41598_2020_57924_MOESM1_ESM.docx]

Likelihood contrasts: a machine learning algorithm for binary classification of longitudinal data

Riku Klén^1,2,+^, Markku Karhunen^1,+^, Laura L. Elo^1,*^

^1^Turku Bioscience Centre, University of Turku and Åbo Akademi University, Finland

^2^Turku PET Centre, University of Turku, Finland

^+^ These authors contributed equally to this work

^*^ Correspondence to laura.elo@utu.fi

Supplementary information


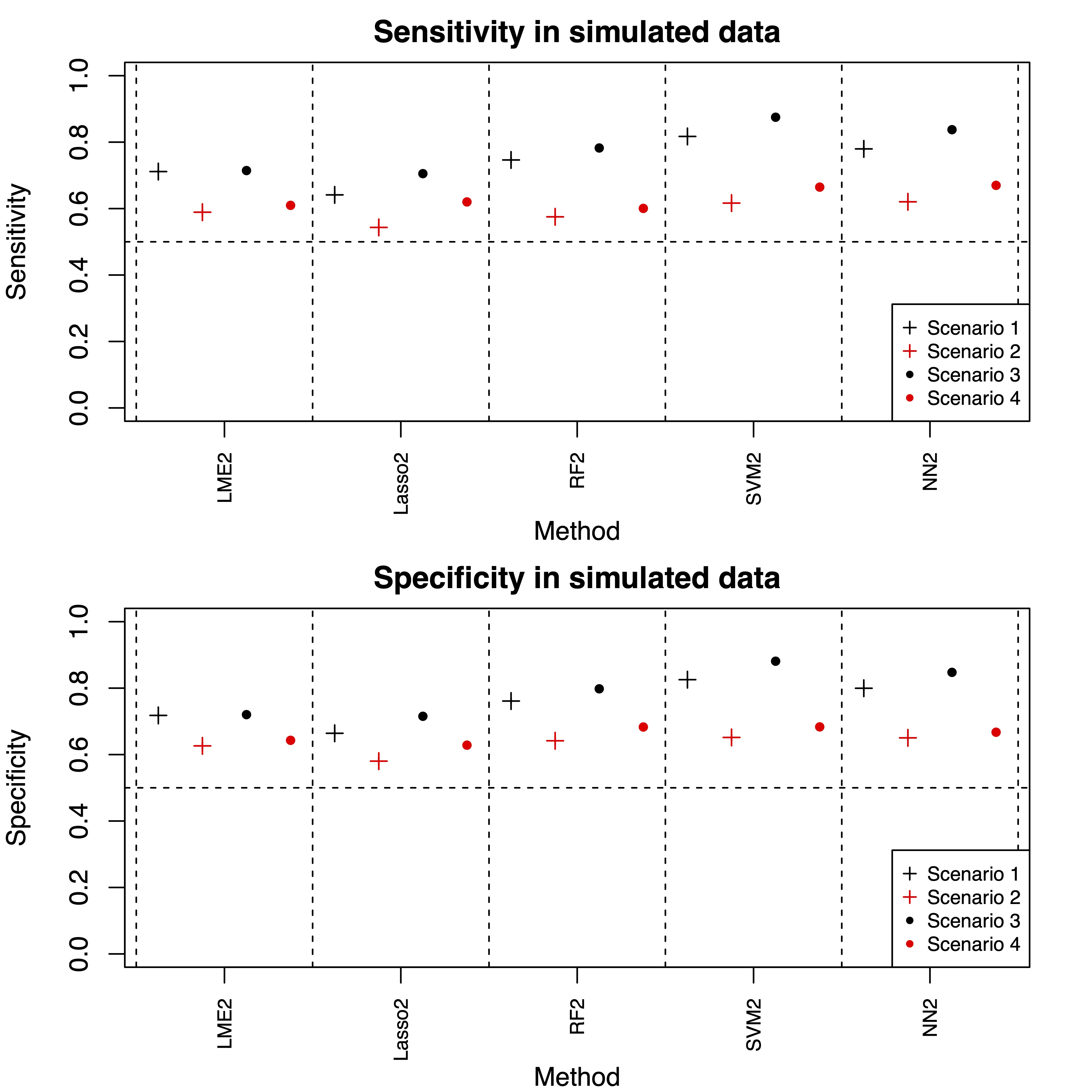


**Supplementary Figure 1. Model performance in simulated data for the methods using each time point as a separate observation.** This figure represents sensitivity and specificity in simulated data for methods LM2, Lasso2, RF2, SVM2 and NN2. The Scenarios refer to data-generating process. The vertical lines around the dots represent standard error, when visible. Sensitivity and specificity have been calculated from 1,000 Monte Carlo replicates.


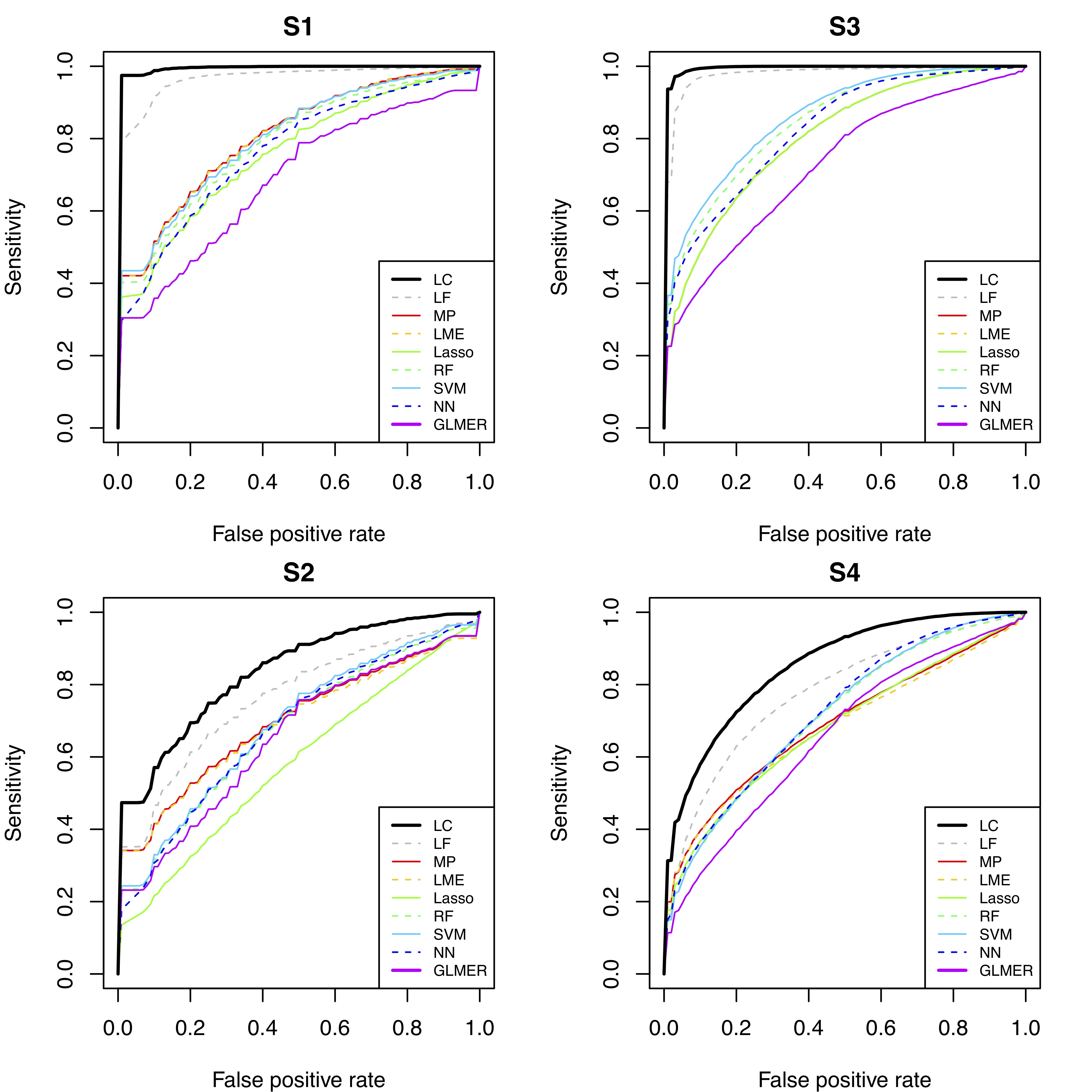


**Supplementary Figure 2. Receiver operating characteristic (ROC) curves in simulated data.** In each simulation scenario S1, S2, S3 and S4, averaged ROC curves were calculated from 1,000 simulation replicates.

**
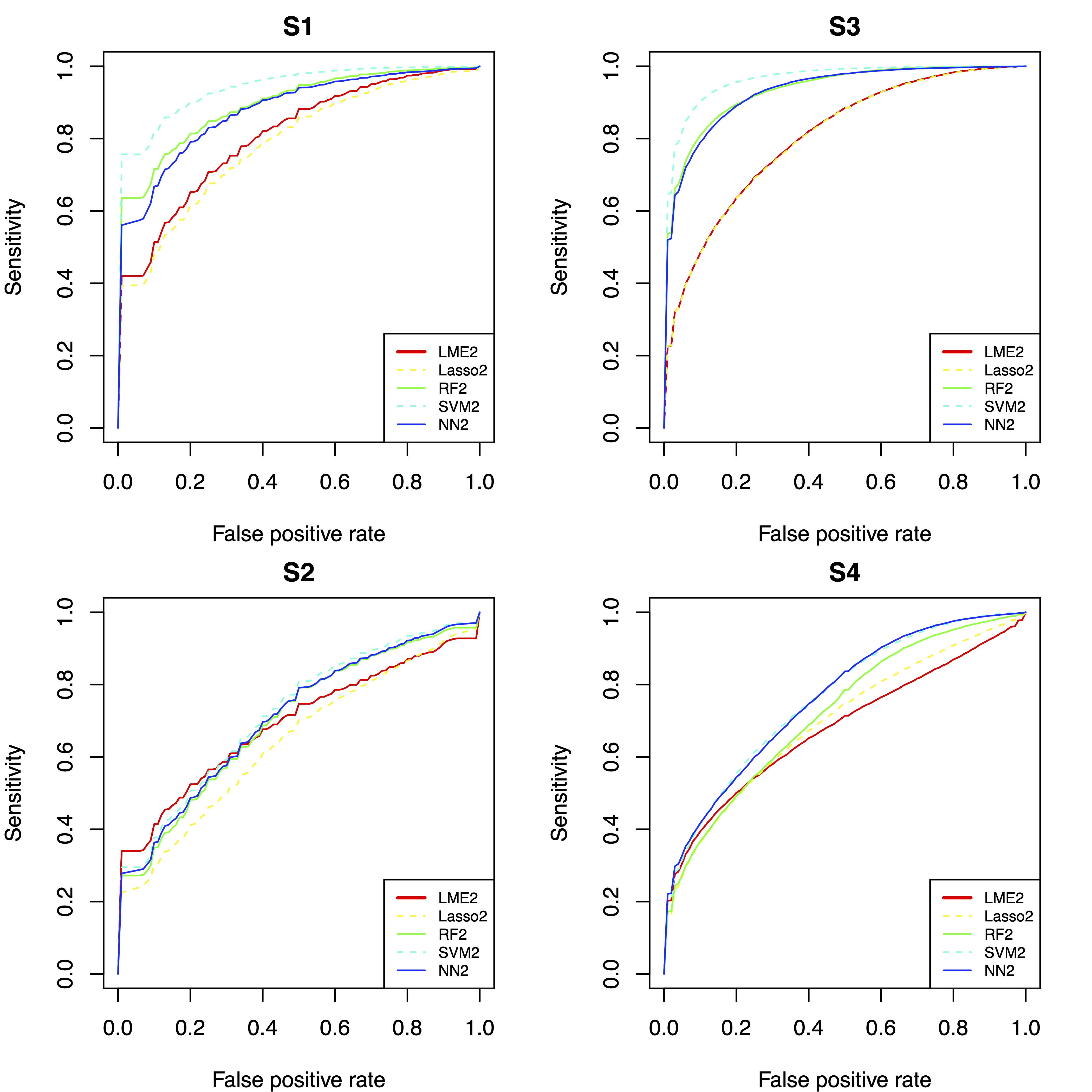
**

**Supplementary Figure 3. Receiver operating characteristic (ROC) curves in simulated data for the methods using each time point as a separate observation.** In each simulation scenario S1, S2, S3 and S4, averaged ROC curves were calculated from 1,000 simulation replicates.


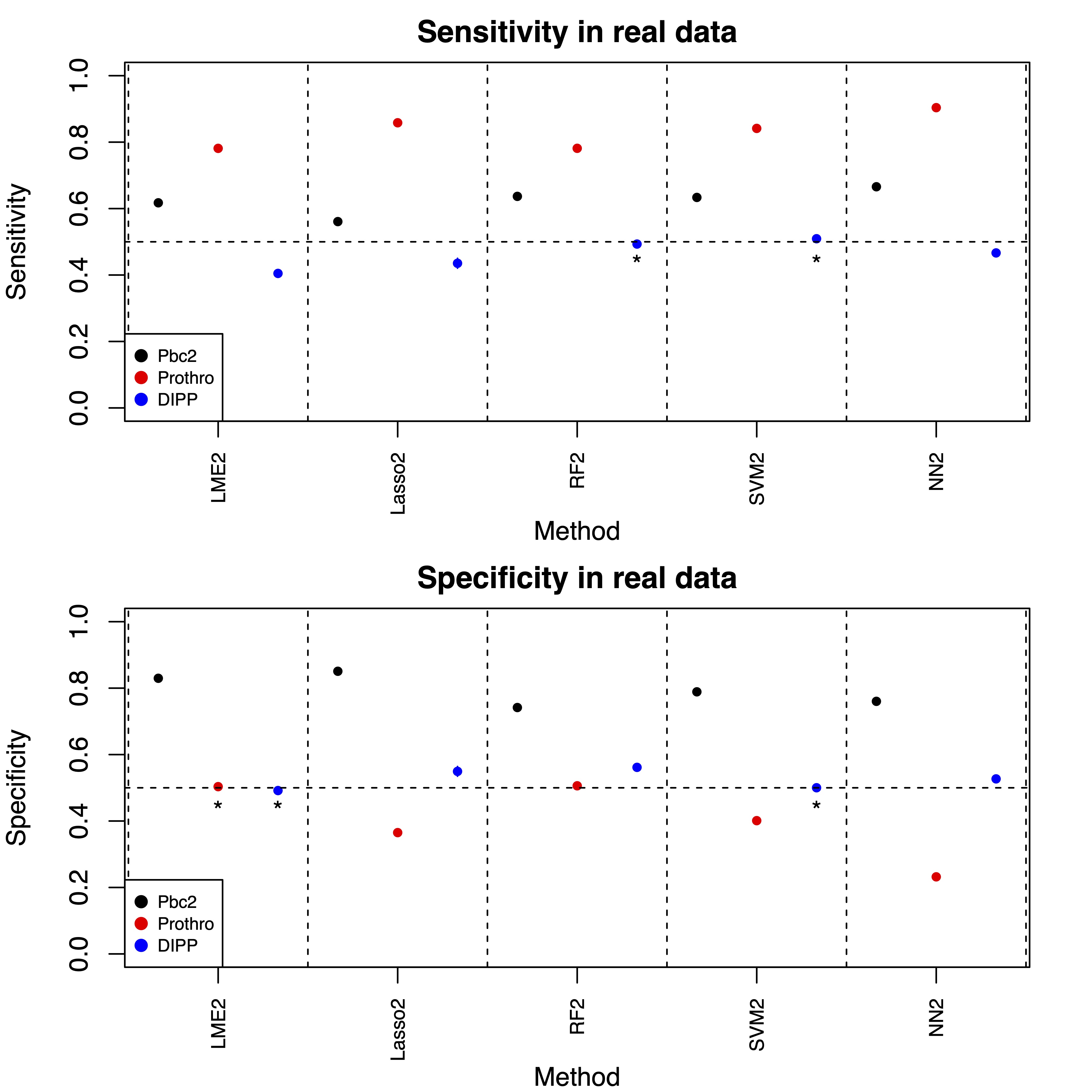


**Supplementary Figure 4. Model performance in real data for the methods using each time point as a separate observation.** Pbc2 and Prothro are clinical data sets, whereas DIPP is a molecular data set from Type 1 Diabetes. The vertical lines around the dots represent standard error, when visible. Sensitivity and specificity have been calculated by using exhaustive cross validation scheme (see Materials). Statistics *not* significant at the false discovery rate level of 0.05 have been indicated by asteriks (*).

**
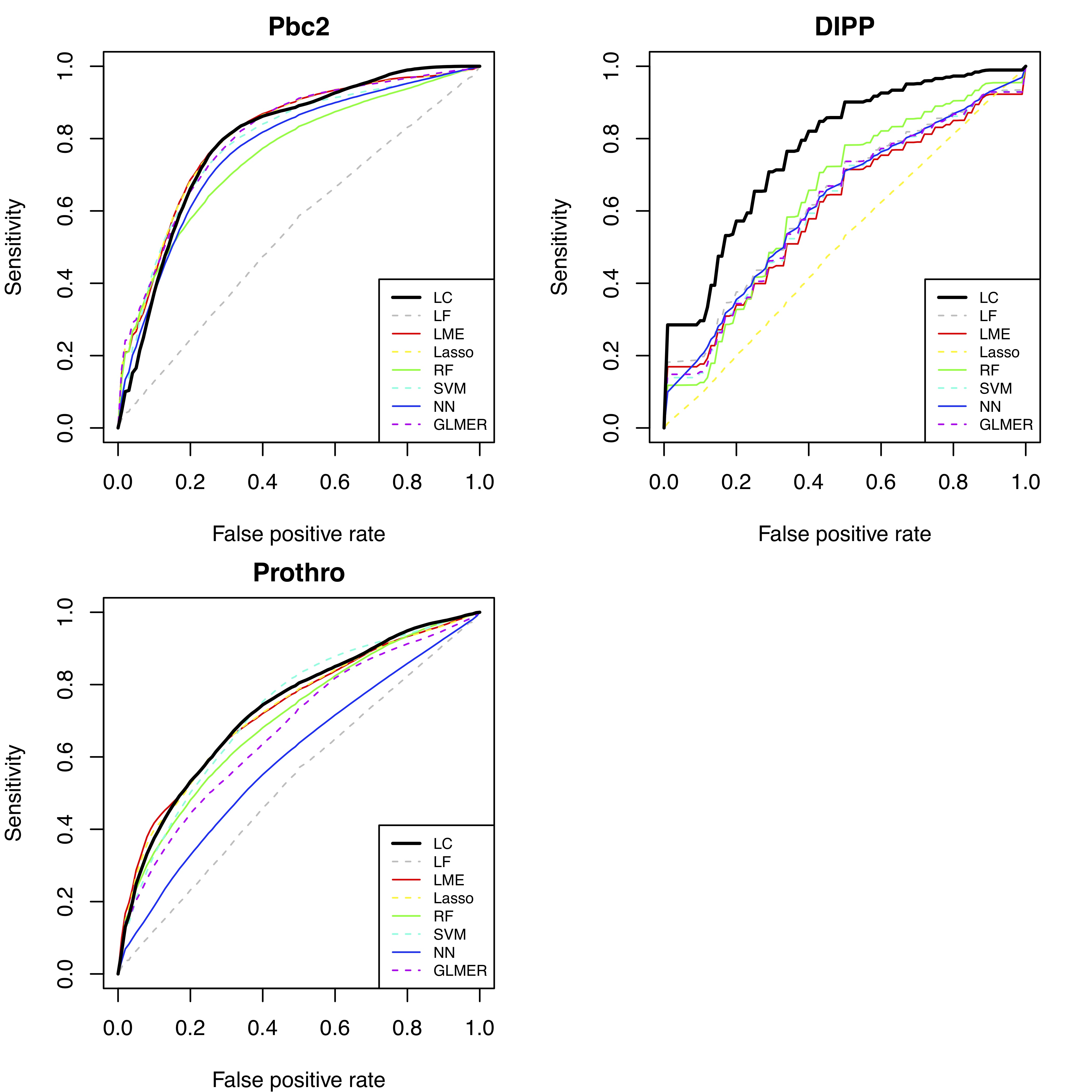
**

**Supplementary Figure 5. Receiver operating characteristic (ROC) curves in the real data sets.** Pbc2 and Prothro were clinical data sets, whereas DIPP was a high-throughput molecular data set on Type 1 Diabetes. Averaged ROC curves were calculated by using an exhaustive cross validation scheme (see Materials).

**
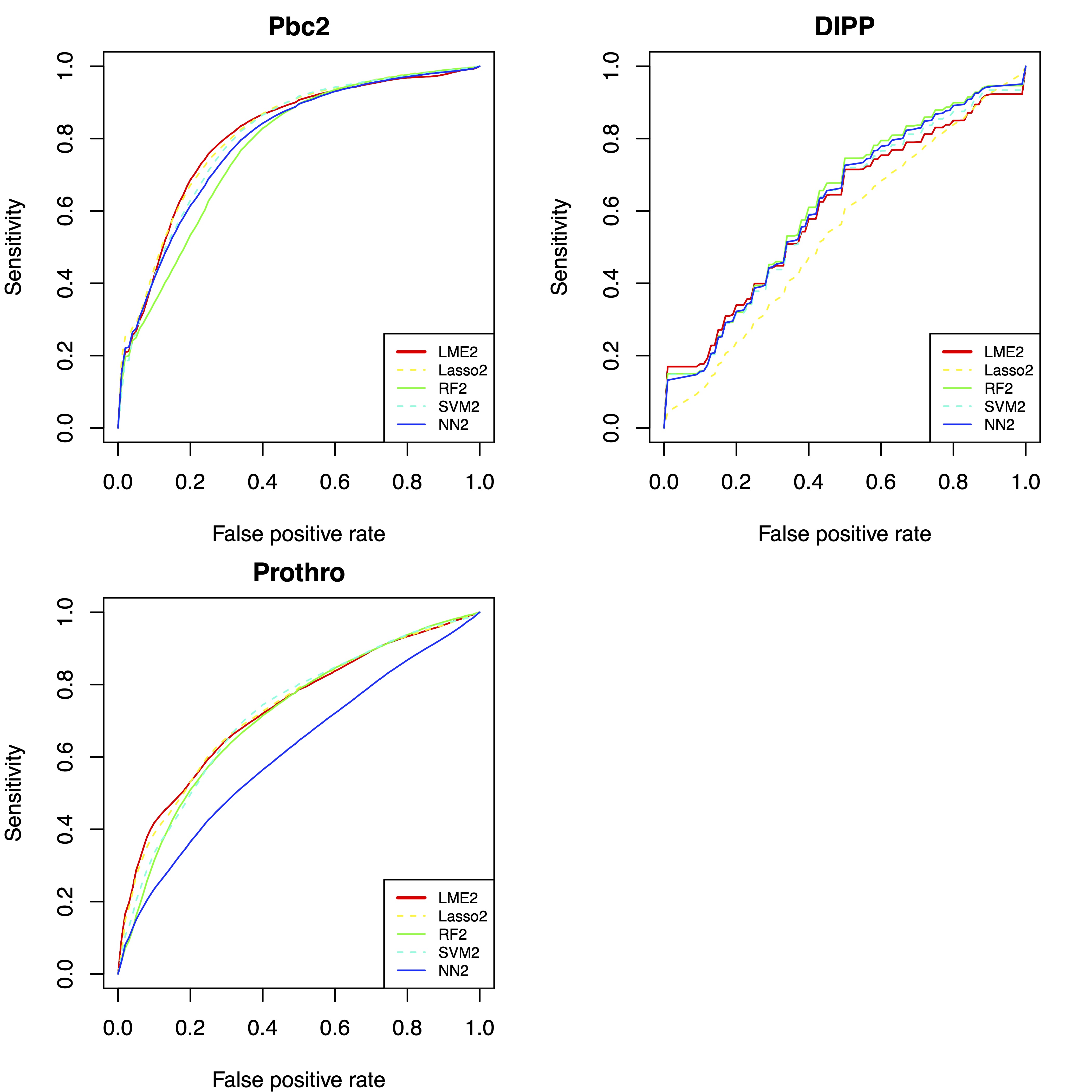
**

**Supplementary Figure 6. Receiver operating characteristic (ROC) curves in the real data sets for the methods using each time point as a separate observation.** Pbc2 and Prothro were clinical data sets, whereas DIPP was a high-throughput molecular data set on Type 1 Diabetes. Averaged ROC curves were calculated by using an exhaustive cross validation scheme (see Materials).

**Supplementary Table 1. Accuracy and F1 scores for the simulated data sets.** Accuracies (Acc) and F1 scores for the simulated data under four different scenarios (S1-S4) were calculated from 1,000 simulation replicates. Standard error in each case was less than 0.005.

|  | S1 | | S2 | | S3 | | S4 | |
| --- | --- | --- | --- | --- | --- | --- | --- | --- |
| Method | Acc | F1 | Acc | F1 | Acc | F1 | Acc | F1 |
| LC | 0.970 | 0.968 | 0.744 | 0.732 | 0.971 | 0.970 | 0.762 | 0.760 |
| LF | 0.938 | 0.935 | 0.690 | 0.666 | 0.947 | 0.947 | 0.709 | 0.702 |
| LME | 0.715 | 0.700 | 0.613 | 0.592 | 0.717 | 0.713 | 0.630 | 0.618 |
| LME2 | 0.715 | 0.699 | 0.606 | 0.586 | 0.717 | 0.713 | 0.627 | 0.614 |
| Lasso | 0.650 | 0.653 | 0.539 | 0.603 | 0.714 | 0.707 | 0.615 | 0.604 |
| Lasso2 | 0.653 | 0.641 | 0.56 | 0.576 | 0.710 | 0.701 | 0.625 | 0.608 |
| RF | 0.715 | 0.694 | 0.615 | 0.581 | 0.747 | 0.738 | 0.643 | 0.624 |
| RF2 | 0.755 | 0.731 | 0.606 | 0.563 | 0.790 | 0.776 | 0.641 | 0.598 |
| SVM | 0.728 | 0.705 | 0.625 | 0.589 | 0.768 | 0.760 | 0.653 | 0.641 |
| SVM2 | 0.822 | 0.807 | 0.633 | 0.598 | 0.878 | 0.875 | 0.673 | 0.655 |
| NN | 0.711 | 0.692 | 0.623 | 0.593 | 0.733 | 0.720 | 0.646 | 0.632 |
| NN2 | 0.791 | 0.775 | 0.635 | 0.604 | 0.842 | 0.838 | 0.668 | 0.653 |
| GLMER | 0.604 | 0.586 | 0.554 | 0.542 | 0.667 | 0.642 | 0.594 | 0.570 |

**Supplementary Table 2. Accuracy and F1 scores in the real data sets.** Accuracies and F1 scores for the real data sets Pbc2, Prothro and DIPP were calculated using an exhaustive cross validation scheme (see Materials). Standard error in each case was less than 0.005.

|  | Pbc2 | | Prothro | | DIPP | |
| --- | --- | --- | --- | --- | --- | --- |
| Method | Acc | F1 | Acc | F1 | Acc | F1 |
| LC | 0.761 | 0.744 | 0.670 | 0.701 | 0.723 | 0.719 |
| LF | 0.474 | 0.396 | 0.499 | 0.637 | 0.465 | 0.520 |
| LME | 0.748 | 0.706 | 0.666 | 0.738 | 0.427 | 0.252 |
| LME2 | 0.733 | 0.672 | 0.667 | 0.736 | 0.388 | 0.350 |
| Lasso | 0.734 | 0.676 | 0.644 | 0.748 | 0.469 | 0.398 |
| Lasso2 | 0.719 | 0.638 | 0.655 | 0.747 | 0.414 | 0.446 |
| RF | 0.697 | 0.656 | 0.649 | 0.710 | 0.576 | 0.512 |
| RF2 | 0.692 | 0.647 | 0.668 | 0.736 | 0.487 | 0.433 |
| SVM | 0.731 | 0.685 | 0.694 | 0.757 | 0.501 | 0.400 |
| SVM2 | 0.717 | 0.665 | 0.660 | 0.746 | 0.451 | 0.424 |
| NN | 0.715 | 0.688 | 0.635 | 0.747 | 0.561 | 0.511 |
| NN2 | 0.716 | 0.683 | 0.631 | 0.744 | 0.465 | 0.407 |
| GLMER | 0.712 | 0.629 | 0.597 | 0.746 | 0.440 | 0.251 |
